# Impact of an Artificial Intelligence-Based Solution on Clinicians’ Clinical Documentation Experience: Initial Findings Using Ambient Listening Technology

**DOI:** 10.1007/s11606-024-08924-2

**Published:** 2024-07-09

**Authors:** J. Luke Galloway, Dominique Munroe, Pamela D. Vohra-Khullar, Christopher Holland, Mary A. Solis, Miranda A. Moore, Reema H. Dbouk

**Affiliations:** 1 Department of Surgery, Emory University School of Medicine, Atlanta, GA USA; 2 Department of Family and Preventive Medicine, Emory University School of Medicine, Atlanta, GA USA; 3 Department of Medicine, Emory University School of Medicine, Atlanta, GA USA; 4 Emory Digital, Emory Healthcare, Atlanta, GA USA

## INTRODUCTION

Escalating levels of clinician burnout have raised significant alarm regarding the well-being of our healthcare providers.^1^ Many studies have correlated the increase in provider burnout with the increasing burden of documentation within the electronic health record (EHR).^2,3^ Artificial intelligence (AI) can improve clinical documentation and positively impact clinician workflow within the EHR.^4,5^ Ambient listening technology is a tool that uses generative AI to generate a clinical note from spoken conversation between clinicians and patients during a scheduled encounter and is available for integration into an EHR’s clinical workflows.^6,7^ We report the impact of a pilot implementation of this tool on clinicians’ documentation experience in the EHR and on overall well-being at Emory Healthcare.

## METHODS

We administered a voluntary web-based survey to a convenience sample of 117 clinicians at Emory Healthcare, a large, urban integrated academic medical institution, at the time of pilot onboarding and 60 days after. The four survey questions addressed three domains: usability, clinician wellness, and patient experience. The follow-up survey contained additional questions on likelihood of recommending this technology, impact on productivity, and intentions for future use. Usability was assessed with a Likert scale with scores ranging from 1 to 5. Well-being and patient experience were assessed by comparing the proportion of negative responses.

Statistical analysis was performed in Stata 14 (StataCorp, LLC, College Station, TX) comparing means of the pre- and post-intervention groups using paired-sample two-tailed *t*-tests, with an alpha of 0.05 determining significance. This study was not deemed to be human subjects research by the Emory University Institutional Review Board.

## RESULTS

The onboarding survey received 117 unique responses. The follow-up survey received 55 unique responses, with an overall response rate of 47%. In total, 31 participants completed both the onboarding and follow-up surveys with 58.1% identifying as male, 64.5% as white, 48.4% between the ages of 20 and 39, mean tenure as 8.4 years, and 32.3% being a primary care clinician (Table 1).

**Table 1:** Distribution of Survey Participant Characteristics

Variable	Onboarding survey (*N*= 117)		Follow-up survey (*N*=55)		Both (*N*= 31)	
	*N* (SD)	Mean (%)	*N* (SD)	Mean (%)	*N* (SD)	Mean (%)
Gender						
Female/woman	59	50.43%	26	47.27%	13	41.94%
Male/man	50	42.74%	27	49.09%	18	58.06%
No response	8	6.84%	2	3.64%	0	0.00%
Race						
Asian	22	18.80%	11	20.00%	7	22.58%
Black or African American	10	8.55%	6	10.91%	3	9.68%
Hispanic/Latino/of Spanish origin	10	8.55%	2	3.64%	1	3.23%
White	66	56.41%	34	61.82%	20	64.52%
Multi-ethnic	1	0.85%	0	0.00%	0	0.00%
No response	8	6.84%	2	3.64%	0	0.00%
Age						
20–39 years old	50	42.74%	24	43.64%	15	48.39%
40–59 years old	46	39.32%	24	43.64%	12	38.71%
60–79 years old	13	11.11%	5	9.09%	4	12.90 %
Current tenure	9.8 (8.3) [0.32–35.69]		9.7 (8.12) [0.41–30.77]		8.4 (7.42) [0.41–30.77]	
Specialty						
Primary care*	29	24.79%	24	43.64%	10	32.26%
Advanced practice professionals†	16	13.68%	9	16.36%	7	22.58%
Other specialties‡	79	67.52%	29	52.73%	21	67.74%

^*^Primary care includes family medicine, internal medicine, and gerontology

^†^Advanced practice professionals includes physician assistants and nurse practitioners

^‡^Other specialties include cardiovascular, clinical psychology, gastroenterology, general surgery, hospice and palliative care, infectious diseases, neurology, obstetrics and gynecology, oncology, orthopedic sports medicine, otolaryngology, pain medicine, physical medicine and rehabilitation, plastic surgery, podiatry, rheumatology, sleep medicine, and urology

**Figure 1:**
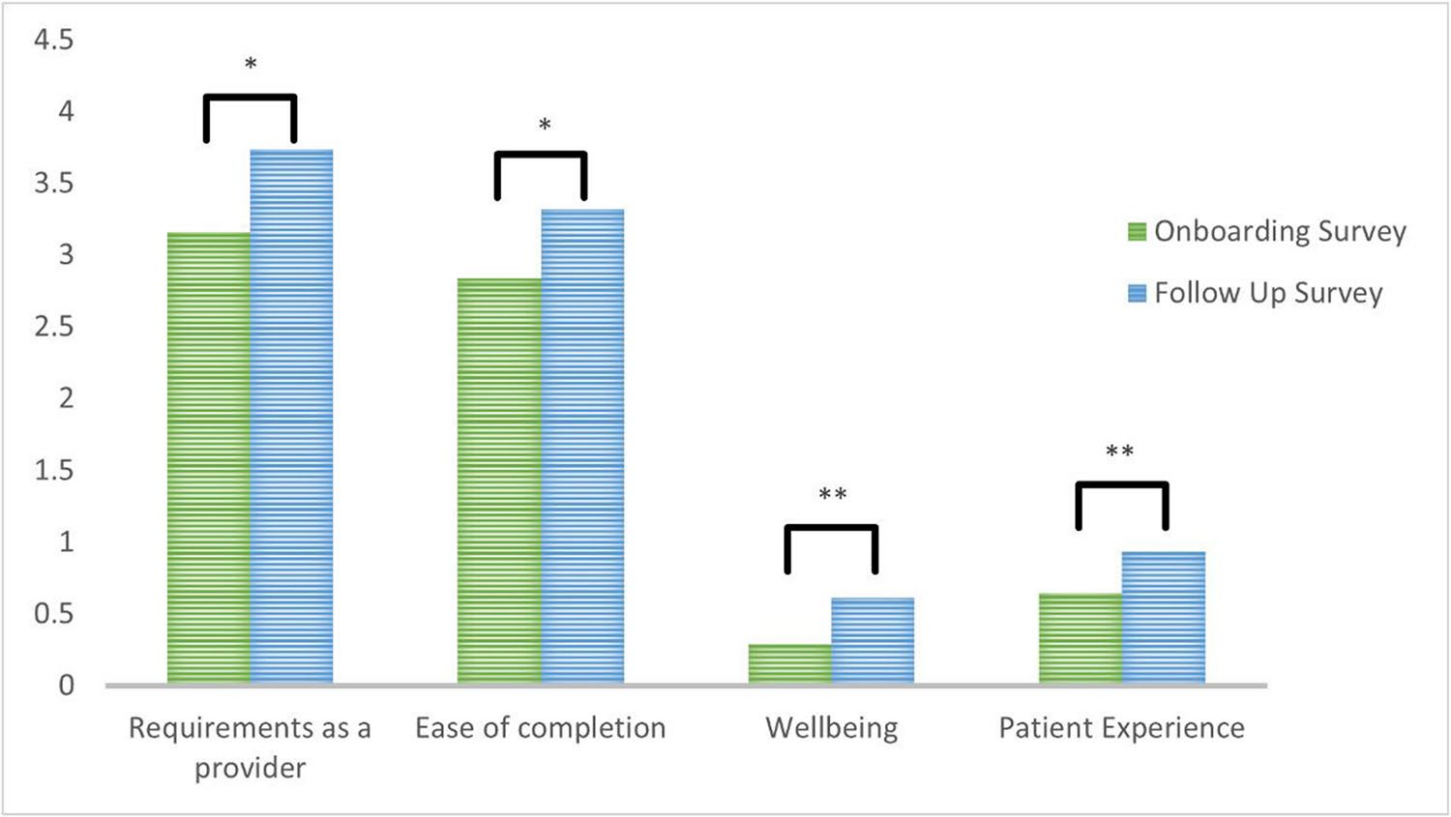
Mean comparison of paired participant survey responses. “My current documentation process meets my requirements as a provider” and “my current documentation is easy for me to complete” were assessed with a Likert scale with scores ranging from 1 to 5. Well-being and patient experience were assessed by comparing the proportion of negative responses. Improvements in each area are indicated by an increase in the score. Statistically significant differences are noted with one asterisk for *p* < 0.05 and two asterisks for *p* < 0.01.

For participants who completed both surveys, when asked if their current documentation process meets their requirements as a provider, 41.9% responded positively compared to 71% post-intervention (*p* = 0.034). Concerning the ease of their current documentation process, 32.3% responded positively compared to 48.4% post-intervention (*p* = 0.023). When asked about the impact of their current documentation process on their well-being, 71% responded negatively compared to 38.7% post-intervention (*p* = 0.010). When asked about the impact of their current documentation process on the patient experience, 35.5% responded negatively compared to 6.5% post-intervention (*p* = 0.005) (Fig. 1). Additionally, 35.5% of participants responded they would be highly likely to recommend this documentation solution to a colleague, 58.1% agreed that it increases their productivity, and 29.0% responded they intend to use it for the majority of their notes.

## DISCUSSION

This generative AI-based ambient listening documentation solution was associated with a significant increase in our participants’ perception of clinical documentation usability and perceptions of positive patient experience. It was also associated with a decrease in the perception of their documentation process having a negative impact on well-being.

Limitations to this study include small sample sizes and a questionnaire change between the pre- and post-intervention surveys, which may have impacted how some questions were interpreted. Specifically, answer choices for questions on well-being and patient experience were changed from “strongly agree – disagree” to “very positive – negative” to improve the questionnaire language.

Generative AI-based ambient listening technology remains promising for improving clinicians’ documentation experience within the EHR. In response to early positive feedback, this technology has already expanded its functionality and soon will be seamlessly integrated into the EHR workflow. The rapid ability to adapt these technologies to the healthcare setting remains an important area of further study.

In addition to reducing the documentation burden for providers, it is hypothesized that use of this technology will also positively impact the patient experience as providers will likely be more focused on the conversation with the patient. Use of this technology may result in more complete and accurate documentation resulting in improved communication with other healthcare providers. More research is needed to explore the impacts of generative AI on the documentation experience and whether this data correlates with reductions in note completion time, after-hours documentation time, and overall EHR time.

## Data Availability

Deidentified data analyzed during the current study is available from the corresponding author upon reasonable request.
